# Supplementary material for: COZOID: contact zone identifier for visual analysis of protein-protein interactions
Source: BMC Bioinformatics. 2018 Apr 6;19:125. doi: 10.1186/s12859-018-2113-6 (PMC5889581; doi:10.1186/s12859-018-2113-6)
Supplement: Supplementary file 2 — Software build. Executable binary file of the software tool. (ZIP 118,784 kb) [file 12859_2018_2113_MOESM2_ESM.zip › cozoid/platform/docs/testng-6.5.1-javadoc.zip!/org/testng/IExtraOutput.html]

IExtraOutput (TestNG)


---


|  |  |  |
| --- | --- | --- |
| **Overview** \| **Package** \| **Class** \| **Tree** \| **Deprecated** \| **Index** \| **Help** | |  |
| **PREV CLASS**   **NEXT CLASS** | **FRAMES**    **NO FRAMES**     **All Classes** |  |
| SUMMARY: NESTED \| FIELD \| CONSTR \| METHOD | DETAIL: FIELD \| CONSTR \| METHOD |  |


---


## org.testng Interface IExtraOutput

**All Superinterfaces:**: java.io.Serializable

---

``` public interface IExtraOutput extends java.io.Serializable ```

This class is used by Reporter to store the extra output to be later
included in the HTML report:
- User-generated report
- Parameter info
Created on Feb 16, 2006

**Author:**
:   Cedric Beust

---

| **Method Summary** | |
| --- | --- |
| `java.util.List<java.lang.String>` | `getParameterOutput()` |

**Method Detail**

### getParameterOutput

```
java.util.List<java.lang.String> getParameterOutput()
```

:   **Returns:**: a List representing the parameters passed to this test method, or an empty List if no parameters were used.


---


|  |  |  |
| --- | --- | --- |
| **Overview** \| **Package** \| **Class** \| **Tree** \| **Deprecated** \| **Index** \| **Help** | |  |
| **PREV CLASS**   **NEXT CLASS** | **FRAMES**    **NO FRAMES**     **All Classes** |  |
| SUMMARY: NESTED \| FIELD \| CONSTR \| METHOD | DETAIL: FIELD \| CONSTR \| METHOD |  |


---
